# The efficacy of serum cell death biomarkers for diagnosing biliary tract cancer

**DOI:** 10.1038/s41598-018-35278-7

**Published:** 2018-11-19

**Authors:** Mitsuru Sugimoto, Kazumichi Abe, Manabu Hayashi, Tadayuki Takagi, Rei Suzuki, Naoki Konno, Hiroyuki Asama, Yuki Sato, Hiroki Irie, Ko Watanabe, Jun Nakamura, Hitomi Kikuchi, Yuichi Waragai, Mika Takasumi, Minami Hashimoto, Takuto Hikichi, Yoshihiro Nozawa, Hiromasa Ohira

**Affiliations:** 1 Department of Gastroenterology, Fukushima Medical University, School of Medicine, Fukushima, Japan; 2 Department of Endoscopy, Fukushima Medical University Hospital, Fukushima, Japan; 3 Department of Pathology, Shirakawa Kousei General Hospital, Shirakawa, Japan

## Abstract

In this study, we determined the efficacy of the cell death biomarker cytokeratin 18 for diagnosing biliary tract cancer (BTC). We recruited 36 patients with BTC (Malignant group) and 45 patients with benign biliary tract disease (Benign group) for this study. We used M30 and M65 as cell death biomarkers. M30 levels indicate apoptosis, and M65 levels indicate both apoptosis and necrosis. M30 and M65 levels were significantly higher in the Malignant group than in the Benign group (142.4 ± 117.0 vs 48.9 ± 71.2 U/l, *P* < 0.001; 1513.3 ± 837.4 vs 882.2 ± 831.2 U/l, *P* = 0.001). The diagnosability of M30 was the highest of the four markers (CEA, CA19-9, M30, M65) (cut-off value: 74.429 U/l, sensitivity: 72.2%, specificity: 77.1%, AUC: 0.771). The sensitivity of M30 (cut-off value: 74.429 U/l) was significantly higher than that of biliary cytology (76% (19/25) vs 12% (3/25), *P* < 0.001), and the accuracy of M30 was significantly higher than that of biliary cytology (78.3% (36/46) vs 52.2% (24/46), *P* = 0.015). The sensitivity of M30 (cut-off value: 74.429 U/l) was significantly higher than that of biliary cytology and brush cytology (72.4% (21/29) vs 24.1% (7/29), *P* < 0.001). In conclusion, cell death biomarkers were increased in patients with BTC, and M30 could efficiently diagnose BTC.

## Introduction

Biliary tract cancer (BTC) is a lethal disease, but diagnosing it is challenging. The diagnostic methods for BTC are biliary cytology, biliary brush cytology, biliary biopsy by endoscopic retrograde cholangiopancreatography (ERCP), and tumour markers (CEA and CA19-9).

The sensitivity of biliary cytology for diagnosing malignant biliary strictures is reported to be 32–57%^1–9^, and the sensitivity of biliary brush cytology is 33–58%^3,4,10,11^. The sensitivity of biliary biopsy for diagnosing malignant biliary strictures is reported to be 36–81%^3,4,7,9,11–13^. All reports except two indicate that the sensitivity of biliary biopsy is no more than 65%. The sensitivity of both biliary brush cytology and biliary biopsy is 61–70.4%^4,11^. In addition, the sensitivity of repeated biliary cytology by endoscopic nasobiliary drainage tube is reported to be 72.4%^14^.

A specific tumour marker for BTC is not available^15^. Although CA19-9 is reportedly increased in up to 85% of patients with cholangiocarcinoma, increased CA19-9 levels can be observed in obstructive jaundice without malignancy. Increased CEA levels are not observed in obstructive jaundice but do occur in 30% of patients with cholangiocarcinoma^16^.

The ERCP procedure and conventional tumour markers are not satisfactory for diagnosing BTC. Interestingly, serum cell death biomarkers have been reported to efficiently diagnose and predict the prognoses of liver diseases^17–22^. Additionally, cytokeratin (CK) 18-associated fragments are related to bile duct loss^23^. Therefore, we hypothesized that serum cell death markers are increased in BTC patients, and we investigated the efficacy of CK 18-associated fragments in diagnosing BTC.

## Results

**Figure 1:**
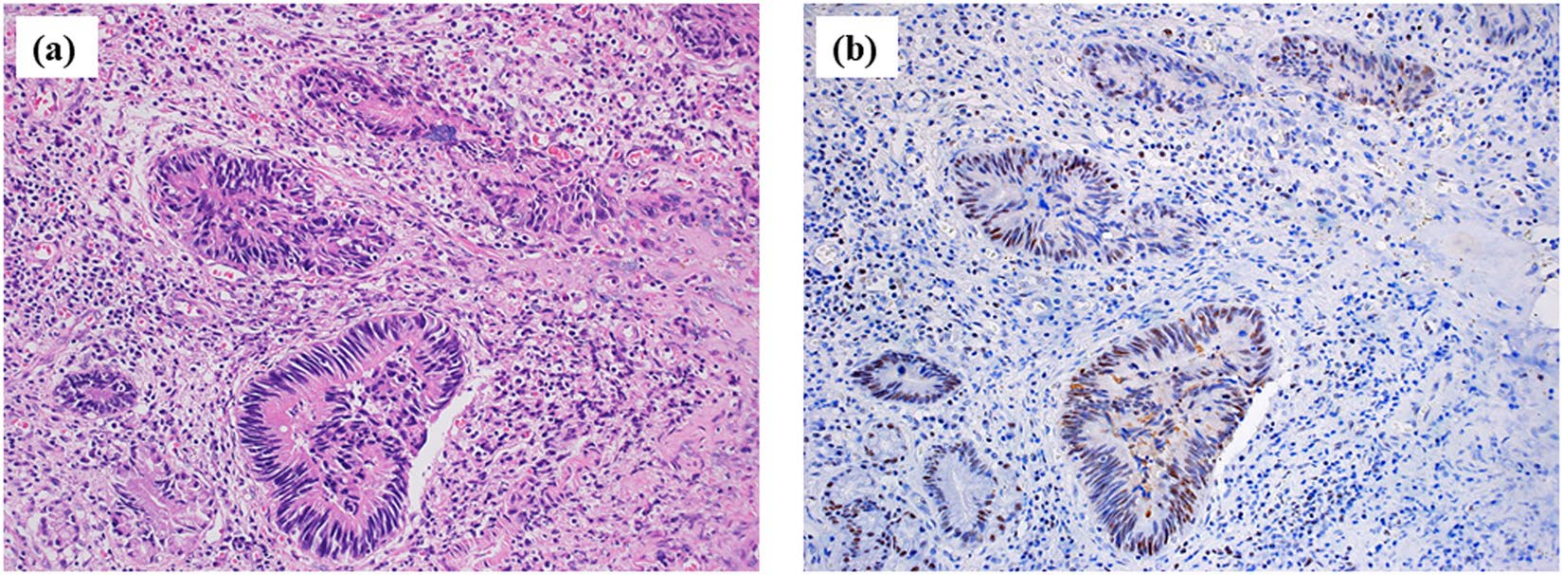
Expression of cytokeratin M30 in a patient with biliary ductal cancer. (**a**) (HE x200), (**b**) (M30 x200). M30 expression was observed in the cytoplasm of biliary ductal cancer cells.

CK M30 was expressed in the cytoplasm of biliary ductal cancer cells (Fig. 1).

**Figure 2:**
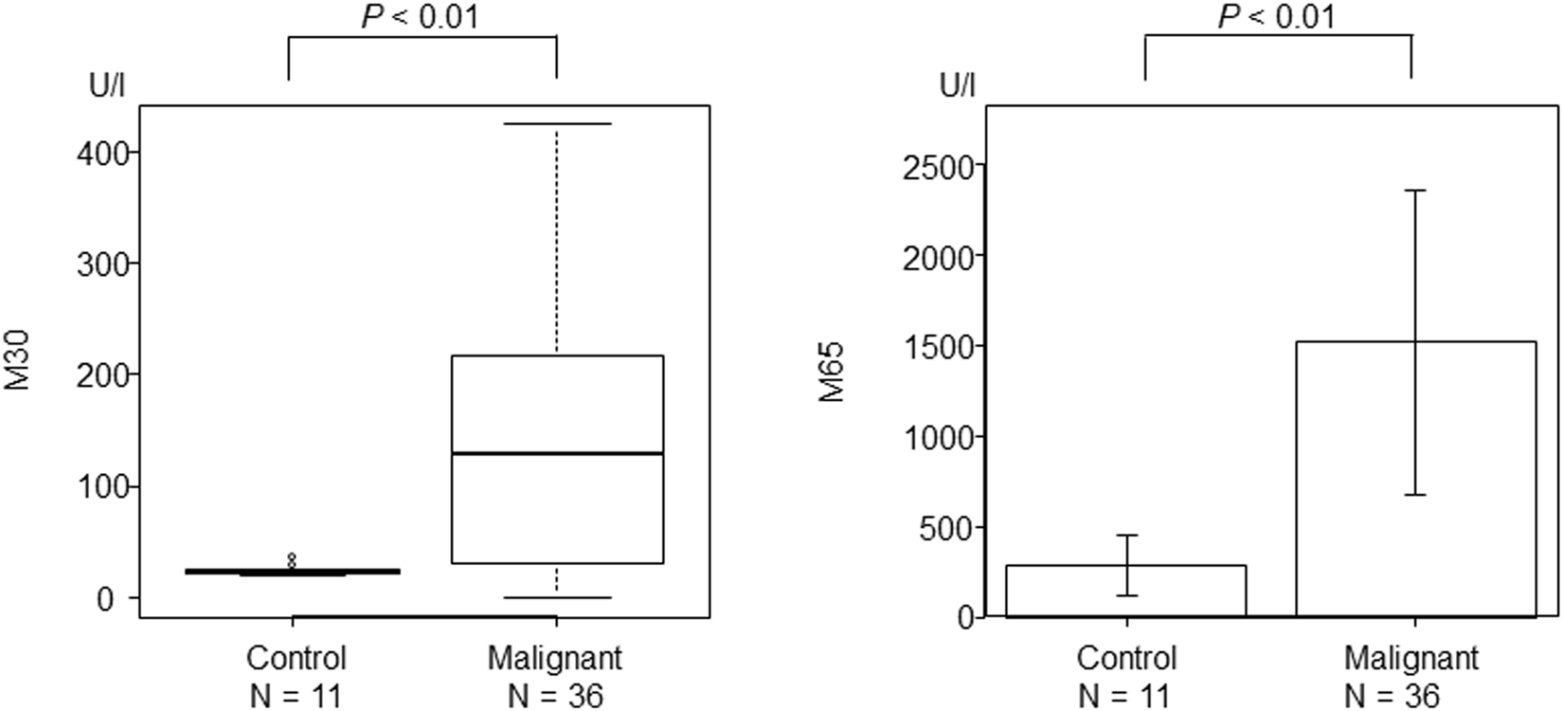
The comparison of cell death biomarkers between the control and Malignant groups. Significantly higher levels of both M30 and M65 were observed in the Malignant group than in the control group.

A comparison of results for cell death biomarkers between the control and the Malignant group are shown in Fig. 2. Significantly higher levels of both M30 and M65 were observed in the Malignant group than in the control group (M30: 21.3 (19.7–37.0) vs 128.3 (0–424.7) U/l, *P* < 0.01, M65: 281.5 ± 166.2 vs 1513.3 ± 837.4 U/l, *P* < 0.01).

The comparative analysis of the patient characteristics and levels of liver transaminases and four markers are shown in Table 1. No variables except for M30, M65, alkaline phosphatase (ALP), and total bilirubin (TB) levels were significantly different between the Malignant group and the Benign group. M30 levels were significantly higher in the Malignant group than in the Benign group (142.4 ± 117.0 vs 48.9 ± 71.2 U/l, *P* < 0.001). Similarly, M65 levels were significantly higher in the Malignant group than in the Benign group (1513.3 ± 837.4 vs 882.2 ± 831.2 U/l, *P* = 0.001). ALP and TB levels were significantly higher in the Malignant group than in the Benign group (ALP 1246.8 ± 1094.0 vs 527.8 ± 425.7 U/l, *P* < 0.001; TB 5.3 ± 6.5 vs 1.6 ± 1.3 mg/dl, *P* = 0.001). M30 showed a weak but significant correlation with the biliary enzymes (ALP or TB) (Table 2), and M65 was significantly correlated with the biliary enzymes.

**Table 1:** Comparison of patient characteristics and markers.

	Malignant (n = 36)	Benign (n = 45)	*P* value
Age (years), mean ± SD	72.6 ± 8.9	71.7 ± 11.8	0.69
Sex (male/female)	26/10	17/10	0.48
UICC stage			
I	9		
II	11		
III	6		
IV	10		
AST (U/l), mean ± SD	124.9 ± 236.9	112.8 ± 215.2	0.81
ALT (U/l), mean ± SD	119.0 ± 143.7	131.0 ± 257.4	0.79
ALP (U/l), mean ± SD	1246.8 ± 1094.0	527.8 ± 425.7	<0.001
TB (mg/dl), mean ± SD	5.3 ± 6.5	1.6 ± 1.3	0.001
CA19-9 (U/ml), mean ± SD	3348.7 ± 8289.6	521.3 ± 2618.7	0.058
CEA (ng/ml), mean ± SD	7.4 ± 18.7	2.7 ± 2.5	0.14
M30 (U/l), mean ± SD	142.4 ± 117.0	48.9 ± 71.2	<0.001
M65 (U/l), mean ± SD	1513.3 ± 837.4	882.2 ± 831.2	0.001

SD, standard deviation; ALP, alkaline phosphatase; TB, total bilirubin.

**Table 2:** Association between cell death biomarkers and ALP or TB.

	ALP		TB	
	r	*P* value	r	*P* value
M30	0.38	<0.001	0.28	0.01
M65	0.55	<0.001	0.69	<0.001

ALP, alkaline phosphatase; TB, total bilirubin.

Malignant lesions and ALP were significantly associated with M30 in the univariate analysis (Table 3); however only malignant lesion was revealed as an independent factor in the multivariate analysis (OR 3.95, 95% CI 1.44–10.80, *P* = 0.008). Malignant lesions, ALP, and TB were significantly associated with M65 in the univariate analysis; however, only ALP and TB were independent factors in the multivariate analysis (ALP ≥ 510 U/l, OR 3.52, 95% CI 1.10–11.3, *P* = 0.03; TB ≥ 1.5 mg/dl, OR 8.82, 95% CI 2.81–27.70, *P* < 0.001).

**Table 3:** Factors that influence M30 or M65.

Univariate analyses of factors that influence M30			
	M30 < 51.69 U/l (n = 40)	M30 ≥ 51.69 U/l (n = 41)	*P* value
Age ≥ 72 years old	18	23	0.38
Sex (male/female)	26/14	28/13	0.82
Malignant lesion	10	26	<0.001
ALP ≥ 510 U/l	14	27	0.007
TB ≥ 1.5 mg/dl	18	24	0.269
**Multivariate analyses of factors that influence M30**			
	**OR**	**95% CI**	***P*** **value**
Malignant lesion	3.95	1.44–10.80	0.008
ALP ≥ 510 U/l	2.33	0.86–6.31	0.10
**Univariate analyses of factors that influence M65**			
	**M65** < **833.3 U/l (n** = **40)**	**M65** ≥ **833.3 U/l (n** = **41)**	***P*** **value**
Age ≥ 72 years old	20	21	1.0
Sex (male/female)	24/16	30/11	0.24
Malignant lesion	11/29	25/16	0.004
ALP ≥ 510 U/l	11	30	<0.001
TB ≥ 1.5 mg/dl	9	33	<0.001
**Multivariate analyses of factors that influence M65**			
	**OR**	**95% CI**	***P*** **value**
Malignant lesion	1.66	0.51–5.46	0.40
ALP ≥ 510 U/l	3.52	1.10–11.3	0.03
TB ≥ 1.5 mg/dl	8.82	2.81–27.70	<0.001

ALP, alkaline phosphatase; TB, total bilirubin.

**Figure 3:**
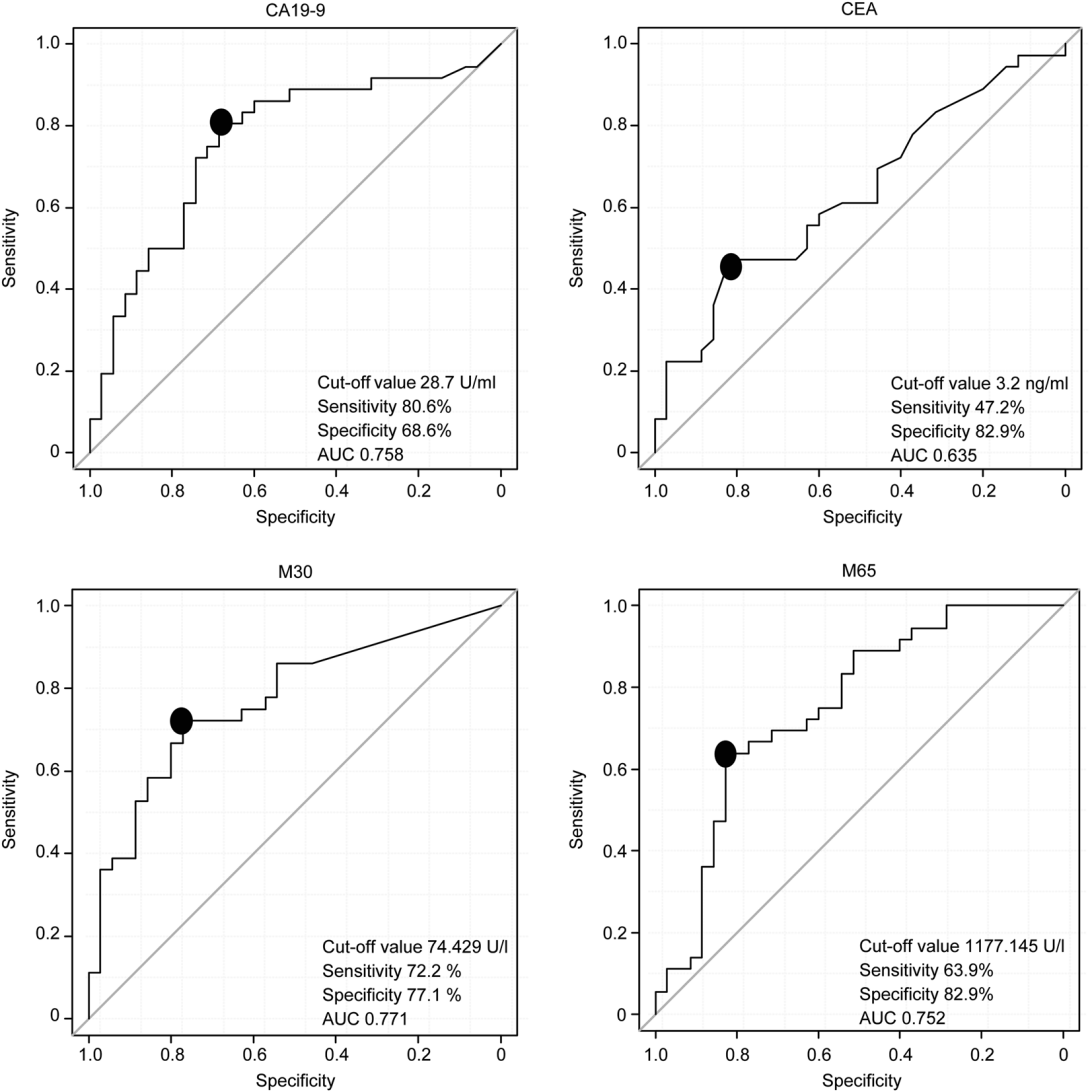
Comparison of four biomarkers for diagnosing biliary tract cancer. *AUC*, area under the curve. Receiver operating characteristic curves of four markers for 71 patients (36: Malignant group, 35: Benign group). The cut-off values, sensitivity, and specificity of M30 for diagnosing biliary tract cancer were 74.429 U/l, 72.2%, and 77.1%, respectively. The AUC (0.771) of M30 was the highest of the four markers.

The comparison of the diagnostic efficacy of these parameters is shown in Fig. 3. The AUC of M30 was the highest of the four markers (cut-off value: 74.429 U/l, sensitivity: 72.2%, specificity: 77.1%, AUC: 0.771).

A comparison of the results for the diagnostic efficacy of M30 (cut-off value: 74.429 U/l) and biliary cytology is shown in Table 4. Biliary cytology was performed for 46 patients. The sensitivity of M30 was significantly higher than that of biliary cytology (76% (19/25) vs 12% (3/25), *P* < 0.001), and the accuracy of M30 was significantly higher than that of biliary cytology (78.3% (36/46) vs 52.2% (24/46), *P* = 0.015).

**Table 4:** Comparison of the malignant diagnosability of M30 and biliary cytology.

	M30 (cut-off value: 74.429 U/l)	Biliary cytology	*P* value
Sensitivity % (n)	76.0 (19/25)	12.0 (3/25)	<0.001
Specificity % (n)	81.0 (17/21)	100 (21/21)	0.11
Accuracy % (n)	78.3 (36/46)	52.2 (24/46)	0.015
PPV % (n)	82.6 (19/23)	100 (3/3)	1.0
NPV % (n)	73.9 (17/23)	48.8 (21/43)	0.07
	**M30** (**cut-off value: 74**.**429 U/l**)	**Biliary cytology or brush cytology**	***P*** **value**
Sensitivity % (n)	72.4 (21/29)	24.1 (7/29)	<0.001
Specificity % (n)	81.0 (17/21)	100 (21/21)	0.11
Accuracy % (n)	76.0 (38/50)	56.0 (28/50)	0.057
PPV % (n)	84.0 (21/25)	100 (7/7)	0.55
NPV % (n)	68.0 (17/23)	48.8 (21/43)	0.14

*PPV*, positive predictive value; *NPV*, negative predictive value.

The comparison of the results for the diagnostic efficacies of M30 (cut-off value: 74.429 U/l), biliary cytology and brush cytology is shown in Table 4. Biliary cytology and brush cytology were performed in 50 patients. Biliary cytology only was performed in 41 patients, and brush cytology only was performed in 4 patients. Both biliary cytology and brush cytology were performed in 5 patients. The sensitivity of M30 was significantly higher than that of biliary cytology or brush cytology (72.4% (21/29) vs 24.1% (7/29), *P* < 0.001).

## Discussion

In this study, we evaluated the efficacy of serum cell death biomarkers for diagnosing BTC. Both serum cell death biomarker levels (M30 and M65) were higher in the Malignant group than in the Benign group. M30 was the most efficient of the four biomarkers (CEA, CA19-9, M30, and M65) and was not as strongly influenced by cholestasis as M65. The sensitivity and accuracy of M30 were higher than the sensitivity and accuracy of biliary cytology for diagnosing BTC. The sensitivity of M30 was higher than that of biliary cytology or brush cytology for diagnosing BTC.

The efficacy of serum M30 and M65 levels, chemotherapy and other treatments in predicting a prognosis for several cancers has been reported^24–32^. In these reports, M30 and M65 levels were reported to be higher in patients with malignant diseases than in patients with benign diseases or healthy controls^26,29,32^. Although the increased serum levels of cell death biomarkers in patients with cholangitis or obstructive jaundice reflect cholangiocyte disruption^30,33^, levels of cell death biomarkers measured in this report were increased to a greater extent in patients with BTC than in patients with benign biliary diseases, such as CBD stones or benign biliary stricture. Therefore, these data suggest that cell death biomarkers are useful for distinguishing BTC from benign biliary diseases.

Though levels of both M30 and M65 were increased in patients with BTC, the malignant diagnosability of M30 was higher than that of M65, possibly because M30 was not as strongly influenced by cholestasis as M65. In past reports, M65 was more strongly correlated with biliary enzymes than M30^20,23^. However, as previously mentioned, M30 is a selective biomarker of apoptotic cell death, and M65 is a marker of both apoptosis and necrosis. However, serum M65 levels are not correlated with histopathological tumour necrosis according to factors other than tumour cell biology^30^. In contrast, serum M30 levels are reported to be correlated with tumour size^28^. The differences between the correlation of M30 and M65 with tumour biology and cholestasis might impact the diagnosability of M30 and M65.

There are some limitations of this study. First, this study was performed with a small number of patients at single institution. We hope to conduct a larger study with similar aims in the future. Second, patients with primary biliary cholangitis (PBC) were not included in this study. Cell death biomarkers have been reported to be higher in patients with PBC than in control subjects^20^. We also hope to conduct a study including patients with PBC in the benign group. Third, the detection ability of the M30 ELISA was different among the various kits. Pimentel C.F. *et al*. compared two M30 ELISA kits and found differences in the detection abilities between the two kits^34^. If the detection ability of an M30 ELISA kit is not satisfactory, trying another kit is necessary. Fourth, cell death markers are increased in other cancers^24–32^. However, biliary cytology and brush cytology are not sufficient for diagnosing whether a biliary lesion is benign or not. In fact, the malignant diagnosability of biliary cytology and brush cytology was inferior to that of M30 in this report. In addition, two BTC cases that were proven to be benign by biliary biopsy had increased serum M30 levels (cut-off value: 74.429 U/l). According to previous reports and this report, serum cell death biomarkers are thought to be efficient for malignant screening. If imaging indicates an abnormality in the biliary tract, M30 will be useful for diagnosing BTC.

In conclusion, cell death biomarkers are increased in patients with BTC, and M30 can efficiently diagnose BTC.

## Methods

### Ethics

This study was approved by the Institutional Review Board of Fukushima Medical University. The methods were performed in accordance with the approved guidelines.

### Patients

We assessed 81 patients with biliary tract disease who visited our hospital between May 2015 and September 2017. Forty-five patients were diagnosed with benign biliary tract diseases or benign biliary strictures (Benign group) (common bile duct (CBD) stones: 33; benign CBD stricture: 10 (autoimmune pancreatitis: 4, chronic pancreatitis: 2, unknown origin: 2, acute pancreatitis: 1, intraductal papillary mucinous neoplasm: (1); Lemmel syndrome: 1; and primary sclerosing cholangitis: (1) (Table 5). The other 36 patients were diagnosed as having malignant BTC (Malignant group) (biliary ductal cancer: 34; gallbladder cancer: (2). The patients provided written informed consent. Patients were diagnosed with malignant biliary diseases by using biliary cytology, biliary brush cytology, biliary biopsy, endoscopic ultrasonography-guided fine needle aspiration, positron emission tomography, or surgery. According to the cytology grades, class IV and V were diagnosed as malignancy. Additionally, we recruited healthy control subjects (N = 11). All patients agreed to participate in this study.

**Table 5:** Biliary diseases and the causes of biliary stricture in the patients.

Malignant (n = 36)		Benign (n = 45)	
Biliary ductal cancer	34	CBD stones	33
Gall bladder cancer	2	CBD stricture	
		Autoimmune pancreatitis	4
		Chronic pancreatitis	2
		Unknown origin	2
		Acute pancreatitis	1
		Intraductal papillary mucinous neoplasm	1
		Lemmel syndrome	1
		Primary sclerosing cholangitis	1

*CBD*, common bile duct.

### Histology and immunostaining

The expression of CK 18 in the cytoplasm of biliary epithelial cells has been reported by Hayashi *et al*.^23^. Therefore, we performed CK M30 immunostaining of a biliary ductal cancer specimen. A mouse monoclonal antibody (M30 CytoDEATH; Roche Applied Science, Mannheim, Germany) was used for M30 immunostaining^35^.

### Measurement of serum cell death biomarkers

Serum samples were collected during hospitalization and were preserved at −80 °C. The serum samples were thawed at room temperature before the serum cell death biomarkers were measured. We assessed CK 18 fragments using a Human Cytokeratin 18-M30 (M30) ELISA kit (CUSABIO, Hubei, China)^23,36,37^, which measured the apoptotic epitopes in the C-terminal domain of CK 18 (amino acids 387–396)^35^. In addition, we used an M65 ELISA kit (VLVbio, Nacka, Sweden), which measured caspase-cleaved CK 18 and uncleaved CK 18. M65 quantitatively measures cell apoptosis and necrosis.

### Patient data

The serum M30 and M65 levels were compared between the control and Malignant groups. Patient characteristics (age and sex) and serum AST, ALT, ALP, TB, CA19-9, CEA, M30, and M65 levels were compared between the Benign and Malignant groups. γ-GTP was not analyzed because at least 20 cases lacked data. The diagnosability of these markers was compared for patients in whom all four markers were measured. The most efficient maker was compared to biliary cytology and brush cytology.

### Statistical analysis

All continuous variables were compared using Student’s t test or Welch’s t test, because the numbers of patients in both groups were sufficient for the analysis, except for the comparison of M30 levels between the control and Malignant groups. M30 levels were compared between the control and Malignant groups using the Mann-Whitney U test. Nominal variables (sex) were compared by Fisher’s exact test. The correlations between cell death biomarkers and biliary enzymes (ALP or TB) were analyzed using the Spearman rank correlation coefficient. The factors that influence cell death biomarkers were analyzed by Fisher’s exact test and multivariate logistic regression. ROC curve analysis was used for diagnosability comparisons. A *P* value < 0.05 was defined as statistically significant. All statistical analyses were performed using the EZR platform (Saitama Medical Centre, Jichi Medical University, Saitama, Japan), which is a graphical user interface for R (The R Foundation for Statistical Computing, Vienna, Austria). EZR is a modified version of the R commander that was designed to perform functions that are frequently used in biostatistics^38^.
